# Temporal trend of disease recognition, treatment paradigm, and clinical outcomes of Crohn disease in Thailand from 2000 through 2017: Is early use of thiopurines beneficial?

**DOI:** 10.1097/MD.0000000000022216

**Published:** 2020-09-18

**Authors:** Julajak Limsrivilai, Satimai Aniwan, Asawin Sudcharoen, Natapat Chaisidhivej, Piyaphan Prueksapanich, Nonthalee Pausawasdi, Phunchai Charatcharoenwitthaya, Supot Pongprasobchai, Sathaporn Manassatit

**Affiliations:** a Division of Gastroenterology, Department of Medicine, Faculty of Medicine Siriraj Hospital, Mahidol University, Bangkok, Thailand; b Division of Gastroenterology, Department of Medicine, Faculty of Medicine, Chulalongkorn University and King Chulalongkorn Memorial Hospital, Bangkok, Thailand.

**Keywords:** behavioral progression, bowel surgery, Crohn disease, hospital-based cohort, immunomodulators, Thailand

## Abstract

The prevalence of Crohn disease (CD) is increasing in Asia, but data from Southeast Asian population are scarce.

The databases of 2 university-based national tertiary referral centers located in Bangkok, Thailand, were retrospectively reviewed for adult patients diagnosed with CD during January 2000 to December 2017. Disease characteristics, diagnosis, treatment, and outcomes were described and compared between the 2000 to 2009 cohort (cohort A) and the 2010 to 2017 cohort (cohort B).

One hundred eighty-two patients (mean age: 46.4 years, 50% male) with 993 patient-years of follow-up were included. Thirteen percent had a history of intestinal resection, but were not diagnosed until disease recurrence. Another 6% were diagnosed at the time of first surgery. There was no improvement in diagnostic proficiency between cohorts. Mesalamine, corticosteroids, thiopurines, and biologics were prescribed in 75.8%, 81.3%, 84.6%, and 13.7% of patients, respectively (*P* > .05 between cohorts). Notably, thiopurines were started earlier in cohort B. Median time to the start of thiopurines was 6.2 and 1.65 months in cohort A and B, respectively (*P* < .01). However, the cumulative 5-year rates of disease behavior progression (*P* = .43), hospitalization (*P* = .14), and bowel surgery (*P* = .29) were not significantly different between cohorts. Subgroup analysis including only patients who required thiopurines showed the early use of thiopurines to be associated with lower risk of intestinal surgery after diagnosis (hazard ratio: 0.30, 95% confidence interval: 0.11–0.85).

Early disease recognition and early introduction of immunomodulators may prevent long-term complications and reduce unnecessary surgery in CD.

## Abstract 2

Supplemental Digital Content is available in the text

## 1 Introduction

Crohn disease (CD) is a chronic inflammatory disease of the gastrointestinal tract that can lead to various disease-related complications if it remains uncontrolled. It has been reported that approximately 80% of CD patients will require some variety of CD-related surgery at some point in their lifetime.^[1]^ As a result of its association with a host of significant health burdens, many population-based CD cohorts have been established to assess the impact of treatments on disease outcomes. These cohorts will continue to help us improve our methods of managing CD, especially since new treatment modalities continue to be introduced.

The prevalence of CD is now increasing in Asian population.^[26,29]^ Although some long-term CD cohorts from Asia have been reported, most of them are from East and South Asia.^[7,9,16,22,28,34,44,47,48]^ Data from Southeast Asia remain scarce with only a few reports in the literature.^[12,25,33]^ Our aim was to describe demographic and clinical characteristics, diagnostic proficiency, treatment pattern, and disease outcomes of CD in Thailand.

In addition, access to biologics is still challenging in many low to middle-income countries, including Thailand. Therefore, thiopurines, which are affordable and widely available, should be optimally utilized. Early introduction of immunomodulators has been proposed to modify disease progression in high-risk patients.^[8]^ Unfortunately, evidence of the benefits of thiopurines in Southeast Asian population is lacking. Therefore, we also aimed to evaluate the effect of the early use of thiopurines on the rate of CD-related surgery.

## 2 Methods

### 2.1 Study design and patients

We retrospectively collected the data of all adult (>18 years) patients diagnosed with CD during January 1, 2000, to December 31, 2017, at Siriraj Hospital or King Chulalongkorn Memorial Hospital, both of which are located in Bangkok, Thailand. Patients lost to follow-up were censored at the date of their last observation. CD was diagnosed based on clinical, endoscopic, and pathologic findings, and the diagnosis was confirmed by clinical and/or endoscopic response to CD treatment. All included patients had at least 6 months of follow-up. The protocol for this study was approved by the Institutional Review Board of both participating hospitals.

### 2.2 Data collection

Demographic and lifestyle data (age, gender, and smoking status), disease characteristics at the time of diagnosis according to Montreal Classification,^[45]^ and medication use data were collected. Medical therapy included 5-aminosalicylates (5-ASA), systemic corticosteroids, immunomodulators (azathioprine, mercaptopurine), and biologics. Early use of immunomodulators or biological therapy was defined as initiation of either of these 2 drugs within 6 months of diagnosis. Diagnosis-related data, including year of diagnosis, duration from presenting symptoms to diagnosis, number of colonoscopies before diagnosis, and anti-tuberculosis treatment before diagnosis, were collected.

Clinical outcomes, defined as disease progression from inflammatory to complicated phenotype (either stricturing or penetrating phenotype), need for intestinal surgery, and CD-related hospitalization(s) after CD diagnosis, were collected. Intestinal surgery included intestinal resection, stoma formation, and percutaneous drainage for concealed perforation. Hospitalizations were considered to be CD-related in cases of CD disease flare or complications of CD treatment, such as opportunistic infections. Elective hospital admissions, such as endoscopy procedures or drug administration, were excluded.

Since biologics was introduced to the treatment of CD in Thailand in 2010 onward and there was an update in the European Crohn's and Colitis Organization (ECCO) guideline in the same year suggesting the early use of azathioprine/mercaptopurine or biologics to be an appropriate treatment option in moderately to severely active CD,^[10]^ the study population was divided into 2 cohorts that represented patients diagnosed before 2010 (2000–2009 - cohort A) and during or after 2010 (2010–2017 - cohort B).

### 2.3 Analysis to evaluate the effect of early introduction of thiopurines on the need for surgery

This analysis included patients who required treatment with thiopurines and took the medication for at least 3 months, which is the duration required for thiopurines to become effective.^[36]^ Patients who underwent surgery at diagnosis or within 6 months after diagnosis, and patients who received thiopurines for post-surgery prophylaxis were excluded. Univariate and multivariate analysis was used to determine the significance of early use of thiopurines on the need for surgery.

### 2.4 Statistical analysis

Continuous data are presented as mean and standard deviation for parametric distributions, and as median and interquartile range for nonparametric distributions. Comparisons between 2 groups were performed using independent *t* test or Mann–Whitney *U* test. Categorical and ordinal data are presented as number and percentage. Comparisons of these types of data were performed using Chi-square test or Fisher exact test. Survival analysis was used to determine the rates of surgery, hospitalization, and disease progression over time. Standard log-rank test was used to compare survival analysis findings between groups. Multivariate Cox proportional hazards regression was used to identify variables significantly associated with the study outcomes. All statistical analyses were performed using SAS Statistics software (SAS, Inc., Cary, NC), and a *P* value < .05 was considered to be statistically significant.

## 3 Results

One hundred ninety-nine patients with CD were identified. Seventeen were excluded due to having less than 6 months of follow-up. The remaining 182 patients (136 from Siriraj Hospital, 46 from King Chulalongkorn Memorial Hospital) were included. Table 1 summarizes baseline characteristics, disease phenotypes, diagnosis and presentations, and treatment of patients in the whole cohort, and compared between cohort A and cohort B. The mean age of patients was 46.4 ± 16.7 years, and 50% were male. Ninety percent of patients had never smoked, and only 5% were current smokers. No significant differences in demographic data between cohorts A and B were observed. The median follow-up time was 4.67 (interquartile range: 2.0–5.7) years, and total follow-up time was 993 person-years.

**Table 1:**
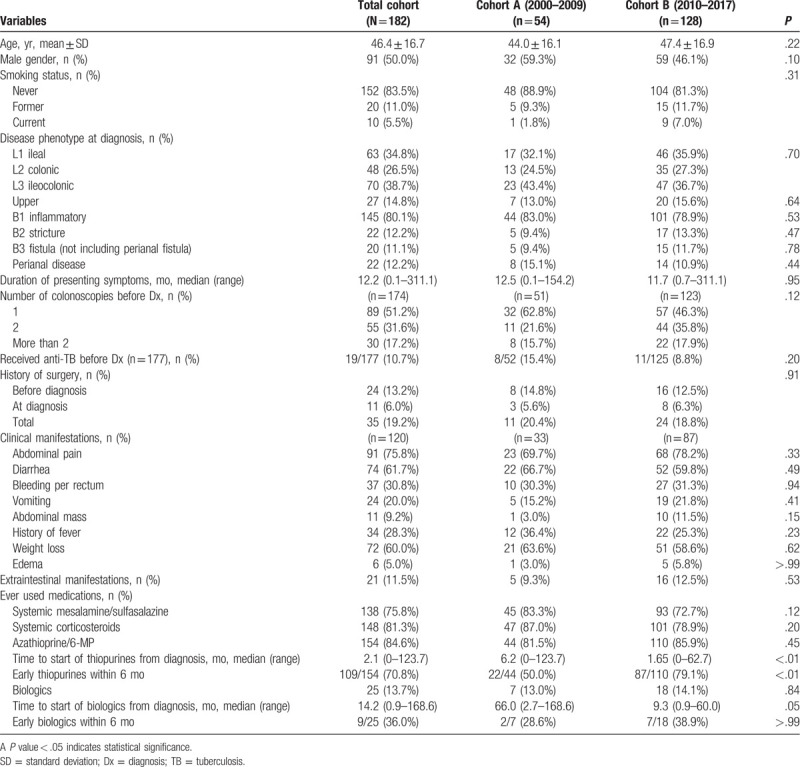
Characteristics, treatment, and outcomes of study patients.

### 3.1 Disease phenotypes, diagnosis, and presentations

At diagnosis, the phenotypic location was 34.8%, 26.5%, and 38.7% for L1, L2, and L3, respectively. Upper gastrointestinal involvement was found in 14.8%. For phenotypic behavior, 80% of patients had inflammatory, 12.2% had stricturing, and 11.1% had penetrating phenotypes. Perianal disease, including perianal fistula and abscess, was found in 12.2%.

The median duration of presenting symptoms prior to diagnosis was 12.2 (range: 0.1–311.1) months. About half of patients required single colonoscopy for diagnosis, 31.6% required 2, and 17.2% required more than 2 colonoscopies. Eleven percent of patients were treated with antituberculosis agents before CD was diagnosed. Thirteen percent of patients had history of bowel resection, but CD was not recognized at the time of that surgery. In those patients, CD was diagnosed upon disease recurrence. Another 6% of patients were diagnosed at the time of surgery. In total, nearly one-fifth of patients had been operated upon before a definite diagnosis of CD was made.

The most common symptom was abdominal pain, which was found in 76%. Other common symptoms included diarrhea and weight loss. Bleeding per rectum and fever was found in about one-third of patients. Extraintestinal manifestations were observed in 11.5%. There was no significant difference observed for disease phenotypes, diagnostic proficiency, or clinical presentations between the 2 cohorts.

### 3.2 Medical treatment

**Figure 1:**
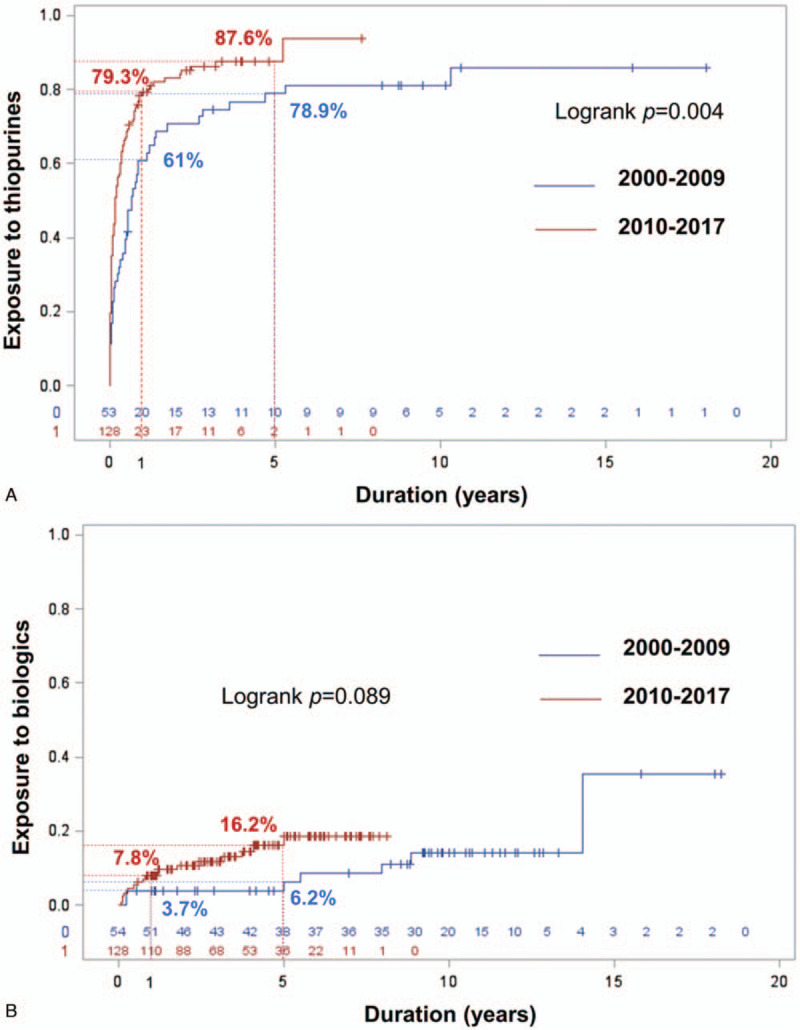
Cumulative exposure to thiopurines (A) and biologics (B) compared between the 2000–2009 cohort and the 2010–2017 cohort.

During follow-up, 138 (75.8%) patients were prescribed 5-ASA, and 148 (81.3%) were prescribed systemic corticosteroids. No significant difference was observed between the 2 cohorts. One hundred fifty-four (84.6%) patients received thiopurines (151 azathioprine, 3 6- mercaptopurine) with no significant difference between groups. However, time to start of thiopurines was significantly shorter in cohort B, as shown in Fig. 1A and Supplementary Table 1, http://links.lww.com/MD/E859. The median time to start of thiopurines from diagnosis was 1.65 (range: 0–62.7) months in cohort B, while it was 6.2 (range: 0–123.7) months in cohort A (*P* < .01). Biologics were used in 13.7% of patients with no significant difference between groups. Nearly all patients received infliximab. Only 1 patient received adalimumab. Similar to thiopurine use, the patients in the cohort B tended to start biologics earlier (Fig. 1B and Supplementary Table 2, http://links.lww.com/MD/E860). The median time to start of biologics was 9.3 months (range: 0.9–60.0) in cohort B, and 66.0 months (range: 2.7–168.6) in the cohort A (*P* = .05).

### 3.3 Behavioral progression

**Figure 2:**
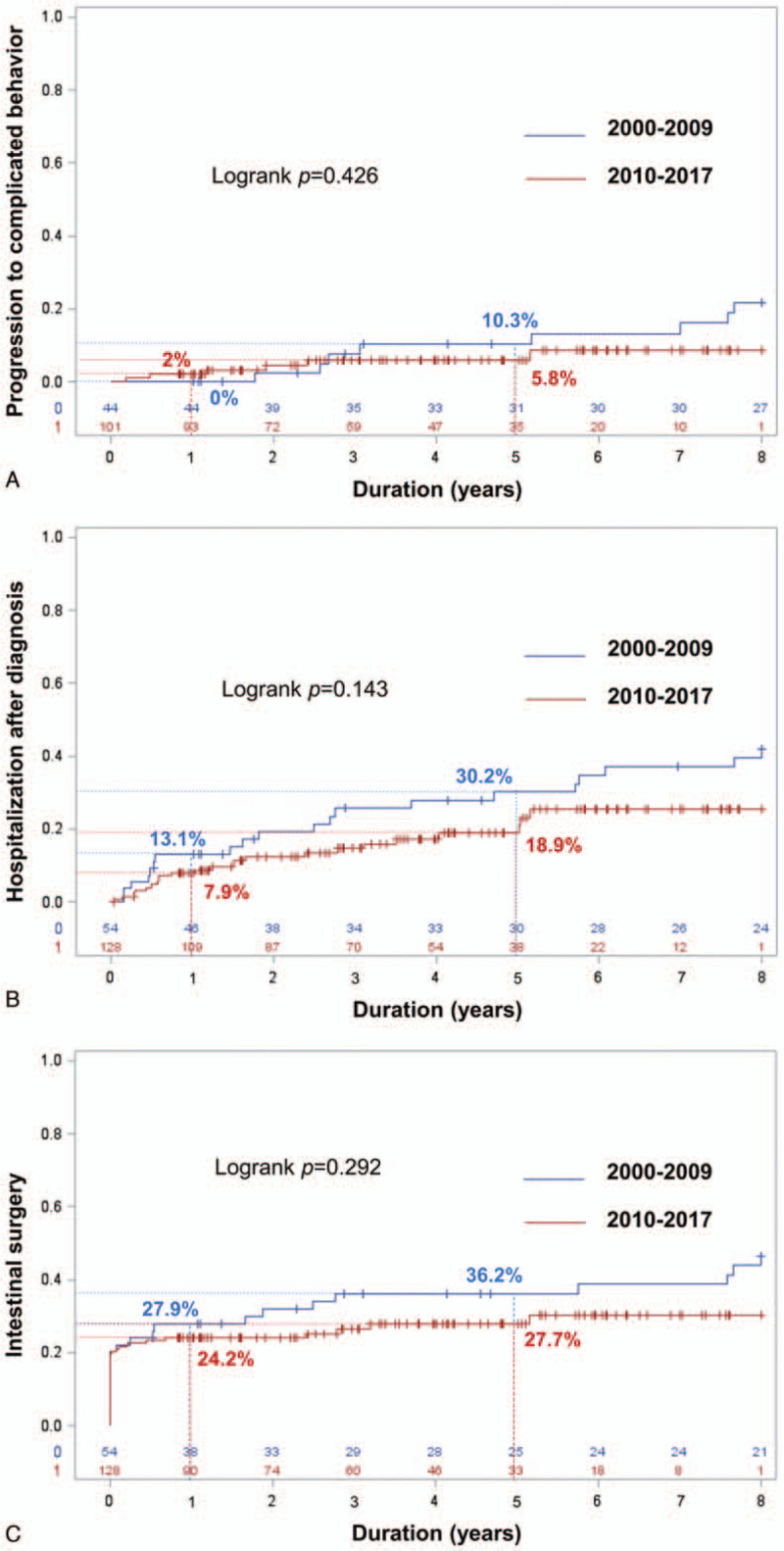
Cumulative rate of disease outcomes. (A) Change from uncomplicated to complicated disease behavior among patients with inflammatory phenotype at diagnosis compared between the 2000 to 2009 cohort and the 2010 to 2017 cohort. (B) Requirement for first hospitalization after CD diagnosis compared between the 2000 to 2009 cohort and the 2010 to 2017 cohort. (C) Requirement for first intestinal surgery compared between the 2000 to 2009 cohort and the 2010 to 2017 cohort.

During follow-up, 14 of 145 patients with inflammatory phenotype at diagnosis (9.7%) developed either bowel stricture or penetration. As shown in Fig. 2A and Supplementary Table 3, http://links.lww.com/MD/E861, there was no difference between cohorts A and B. The cumulative rates of behavior change to complicated behavior at 1 year and 5 years were 0% and 10.3% for cohort A, and 2.0% and 5.8% for cohort B, all respectively (*P* = .43).

### 3.4 First hospitalization after diagnosis

During follow-up, 43 (23.6%) patients required at least 1 hospitalization in 933 person-years. As shown in Fig. 2B and Supplementary Table 4, http://links.lww.com/MD/E862, there was a trend toward a lower number of patients requiring at least 1 hospitalization in cohort B, but the difference between groups did not achieve statistical significance. The cumulative rates of first hospitalization at 1 year and 5 years were 13.1% and 30.2% for cohort A, and 7.9% and 18.9%% for cohort B, all respectively (*P* = .14).

### 3.5 First intestinal surgery

During follow-up, 58 (31.9%) patients required at least 1 intestinal surgery in 933 person-years. As mentioned above, 24 (13.2%) patients had undergone surgery before CD diagnosis, and 11 (6.0%) patients underwent surgery at the time of diagnosis. Among these 35 patients, 4 patients required a second operation. The types of first operations are described in Table 2. As shown in Fig. 2C and Supplementary Table 5, http://links.lww.com/MD/E863, the cumulative rates of surgery at 1 year and 5 years were 27.9% and 36.2% in cohort A, and 24.2% and 27.7% in cohort B, all respectively (*P* = .29). After excluding the surgeries before and at diagnosis, the cumulative rate of bowel resection surgery after CD diagnosis at 1 and 5 years was 5.5% and 13.4%, respectively. Significant difference between the 2 cohorts was not observed (*P* = .69).

**Table 2:**
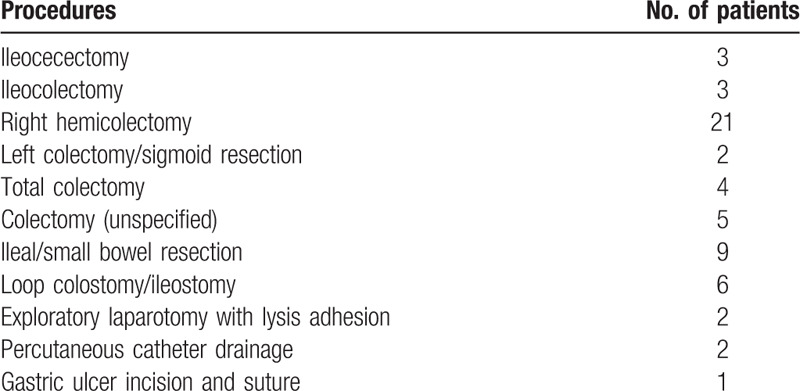
Types of intestinal surgery in this cohort.

### 3.6 Subgroup analysis to evaluate the benefits of early use of thiopurines on the need for surgery

**Figure 3:**
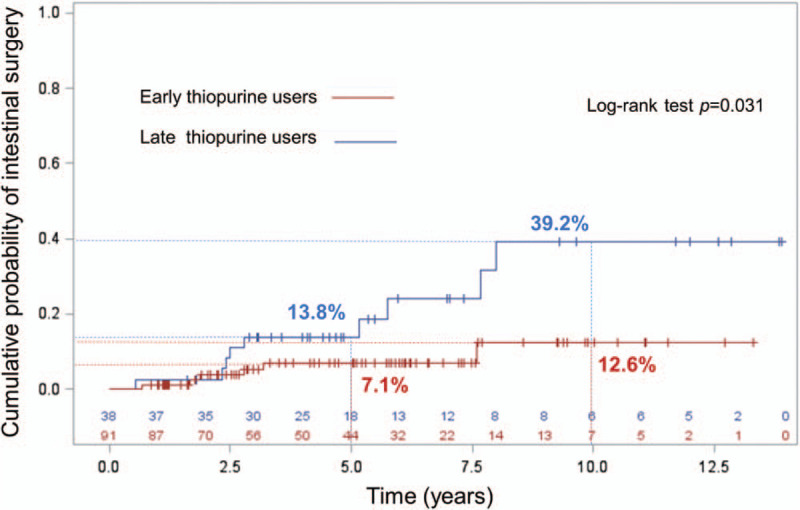
Cumulative rate of requirement for first intestinal surgery after diagnosis compared between patients who started thiopurines within 6 months after diagnosis and those who started thiopurines at 6 months or longer after diagnosis.

Of 182 patients, 19 patients who underwent surgery at the time of diagnosis or within 6 months after diagnosis and who received thiopurines for postoperative prophylaxis were excluded. Of the remaining, 24 who did not use thiopurines and 10 who took thiopurines for less than 3 months were excluded. A total of 129 patients were included in this analysis. Ninety-one (70.5%) patients started thiopurines within 6 months after diagnosis. The mean dosage of azathioprine was 1.6 milligram per kilogram of body weight per day in both groups. Adherence to thiopurines was not significantly different between groups. Specifically, 83.5% in the early use, and 84.2% in the late use group, took thiopurines until their last observation (*P* = .881). The early use group had significantly higher isolated ileal involvement and tended to have more penetrating disease (Supplementary Table 6, http://links.lww.com/MD/E864). The unadjusted rate of CD-related intestinal surgery was significantly lower in the early use group, as shown in Fig. 3. Univariate and multivariate analysis for factors that predict CD-related intestinal surgery is summarized in Table 3. After adjusting for age, gender, disease location, disease behavior, perianal involvement, history of previous surgery, use of biologics, and follow-up time, the early use of thiopurines was found to be an independent protective factor against surgery [hazard ratio (HR): 0.30, 95% confidence interval (95% CI): 0.11–0.85, *P* = .024], while upper GI tract involvement was identified as an independent predictor of CD-related intestinal surgery with a HR of 3.37 (95% CI: 1.14–10.00, *P* = .029).

**Table 3:**
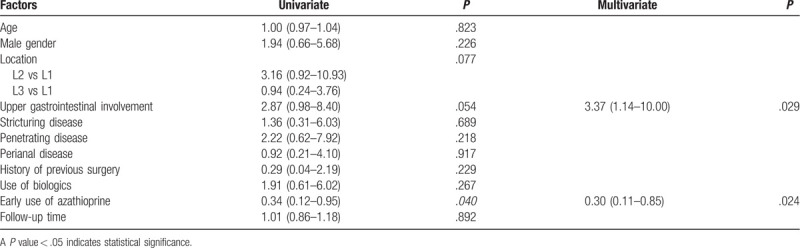
Univariate and multivariate analysis for factors that independently predict Crohn disease-related intestinal surgery in patients who required thiopurine therapy.

## 4 Discussion

CD is increasing in Asia, and the data being reported from Asian CD cohorts are also increasing. However, data from Southeast Asia remain limited. Our cohort is the first long-term follow-up cohort describing the characteristics, phenotypes, manifestations, diagnosis, treatment, and outcomes of CD in Thailand.

The mean age of patients in this study was 46.4 ± 16.7 years. Patients in other cohorts from both Asia and Western countries appear to have been a little younger at diagnosis than our patients. The reported mean or median age at diagnosis in those studies was mostly within the range of 20 to 40 years.^[3,6,9,12–14,16,20,22,25,27,28,30,34,40,41,44,48–50]^ There was no gender preference in our cohort, which is similar to many cohorts from Western countries,^[3,20,31,35,41]^ but most cohorts from Asia reported male predominance at 60% to 70%.^[6,9,13,15,22,27,28,30,34,44,48,49]^ The locations of disease at diagnosis in our cohort were L3 (40%), L1 (35%), and L2 (25%). The majority of cohorts from Asia also showed L2 to be the least likely location of involvement; however, more colonic involvement was found in Western cohorts.^[3,6,13,16,20,27,34,40,41,44,48]^ Similar to other studies, most of the patients in our study had inflammatory phenotype at diagnosis.

Diagnosis of CD remains a challenge in Thailand. The time from onset of symptoms to diagnosis was about 12 months in our cohort. Although this duration is not nearly as long as the 3-year duration reported from India in 2009,^[9]^ it is much longer than the median time to diagnosis of 3 months reported from Hong Kong and China in more recent years.^[22,28]^ The median time to diagnosis in Europe was recently reported to be 4 months.^[3]^ Furthermore, in contrast to the study from China that reported a dramatic decrease in the time to diagnosis from 79.4 months before 2010 to 3.1 months in 2015,^[22]^ the diagnostic time in our study was stable between the before and after 2010 groups. The reasons that we most suspect are physician unawareness of CD and/or physician difficulty differentiating CD from intestinal tuberculosis (ITB). Physician unawareness is evidenced by the significant proportion of patients (13%) who were not diagnosed with CD even though they were operated upon. This rate was not different between cohort A (14.8%) and cohort B (12.5%). In addition, ITB is difficult to differentiate from CD. Eleven percent of patients in the present study received anti-tuberculosis therapy before diagnosis. This rate is, however, lower than the 20% to 40% rates reported from India and South Korea.^[9,16,48]^ Interestingly, there was a trend, although nonsignificant, toward the rate of anti-tuberculosis therapy decreasing before diagnosis in the later period. About 9% of patients in cohort B received anti-tuberculosis therapy before diagnosis, while the rate was 15.4% in cohort A (*P* = .20). We postulate that this could be related to the increased use of scoring systems designed to differentiate ITB from CD in the later study period.^[21,24]^

The medical treatments for CD in this cohort were mainly 5-ASA, glucocorticoids, and thiopurines. Each of these 3 medications were taken at some point during the study period by about 80% of patients. Biologics use was quite limited in this study because biologics are not yet reimbursed by the main health care schemes in Thailand. As such, only 14% of patients were treated with biologic therapy. These proportions of medications used are comparable to those reported from many cohorts from Asia with reported rates of mesalamine, glucocorticoids, thiopurines, and biologics use of 70% to 98.9%, 35% to 100%, 29% to 92.6%, and 0% to 33%, respectively.^[9,12,13,15,27,28,33,48]^ Comparable rates of immunomodulator and biologic use were also reported from pre-2010 Western cohorts with rates of 25% to 64% for immunomodulators, and 0% to 32% for biologics.^[14,20,31,35,40]^ Interestingly, there was a significant trend toward early initiation of immunomodulators and biologics in the later study period. The same pattern was also observed in cohorts from Western countries and South Korea.^[14,20,31,34,40,50]^ Notably, the time of initiation of immunomodulators in our cohort was quite early since the overall cumulative rate of use was 74% and 86% at 1 and 5 years after diagnosis, respectively. Other cohorts that included patients from the 1990s to the early 2000s reported lower cumulative rates of use of 22.2% to 34.8% at 1 year, and 37.7% to 46.2% at 5 years.^[20,40,50]^ (Supplementary Table 7, http://links.lww.com/MD/E865)

Disease outcomes were evaluated by rate of change in disease behavior from inflammatory phenotype to complicated behavior, rate of hospitalization, and rate of surgery. The incidence of these outcomes in this study seemed to be lower than previously reported, and was not significantly different between cohort A and cohort B. The cumulative rate of disease behavior progression at 5 years was 7.6%. This rate is lower than the rates reported from Europe^[14,20]^ and the United States^[35]^ for which disease behavior progression was about 20% at 5 years after diagnosis. Hospitalization after diagnosis was observed in 23.6% of patients during the follow-up period in our cohort. This rate is lower than that reported from Canada, which was 25% per year.^[2]^ In the present study, the cumulative rate of surgery at 1 and 5 years after diagnosis was 25.3% and 30.4%, respectively. This overall 5-year cumulative rate of surgery is comparable with that reported from many cohorts (20–40%) during the same era from both Asian and Western countries.^[3,13,16,20,28,31,34,40,44,48]^ Interestingly and as mentioned above, many operations in this cohort were performed before or at diagnosis. The cumulative rate of surgery at 5 years when surgery after diagnosis was exclusively considered was only 13.4%. Apart from differences in race, we hypothesize that the low prevalence of smoking, which was reported to worsen CD,^[46]^ and the high rate of early use of immunomodulators may account for the low rate of disease progression, hospitalization, and surgery after diagnosis in our cohort.

The benefits of early use of immunomodulators in reducing CD-related surgery are still being debated. Even though some observational studies showed no benefits,^[4,19,37,39,42]^ 1 meta-analysis^[5]^ and other observational studies did show benefit of early use in reducing CD-related surgeries.^[11,17,18,20,23,32,34,38,40,43]^ In this cohort, we performed subgroup analysis to determine this effect. To ensure the benefits of thiopurines, only those who took the medicine for at least 3 months were included. Patients who started thiopurines within 6 months, but after surgery were excluded. This analysis demonstrated that early use of thiopurines was an independent protective factor against CD-related intestinal surgery.

This study has some limitations. First, our data are referral hospital-based data that was retrospectively reviewed. In addition to the known data-related shortcomings of retrospective study, our study could be vulnerable to bias in favor of more severe disease as our 2 study centers are routinely referred cases that are thought not to be manageable in other care settings. Second, we did not have essential details specific to clinical scores and endoscopic severity scores that would have provided more insight into disease severity and progression. Last, the sample size was small and insufficient to generalize conclusions to other populations. This is attributed to the low prevalence of CD in Thailand.

## 5 Conclusion

This study highlights the importance of early disease recognition in a low prevalence area and early introduction of immunomodulators in CD, which could help to prevent long-term complications and reduce unnecessary surgery.

### 5.1 Uncited reference

^[36]^.

## Acknowledgment

The authors gratefully acknowledge Kevin P. Jones (Siriraj Medical Research Center, Faculty of Medicine, Siriraj Hospital, Mahidol University, Bangkok, Thailand) for language editing.

## Author contributions

Julajak Limsrivilai: Study concept and design, data acquisition, data analysis and interpretation, and drafting of the manuscript

Satimai Aniwan: Study concept and design, data acquisition, critical revision of the manuscript

Natapat Chaisidhivej, Asawin Sudcharoen, Piyapan Prueksapanich: Data acquisition

Phunchai Charatcharoenwitthaya, Nonthalee Pausawasdi, Supot Pongprasobchai, Sathaporn Manassatit: Critical revision of the manuscript

**Conceptualization:** Julajak Limsrivilai, Satimai Aniwan.

**Data curation:** Julajak Limsrivilai, Satimai Aniwan, Asawin Sudcharoen, Natapat Chaisidhivej, Piyaphan Pruksapanich.

**Formal analysis:** Julajak Limsrivilai.

**Investigation:** Julajak Limsrivilai.

**Methodology:** Julajak Limsrivilai.

**Project administration:** Julajak Limsrivilai.

**Resources:** Julajak Limsrivilai, Satimai Aniwan.

**Software:** Julajak Limsrivilai.

**Supervision:** Phunchai Charatcharoenwitthaya.

**Writing – original draft:** Julajak Limsrivilai.

**Writing – review & editing:** Julajak Limsrivilai, Satimai Aniwan, Nonthalee Pausawasdi, Phunchai Charatcharoenwitthaya, Supot Pongprasobchai, Sathaporn Manassatit.

## Abbreviations

Abbreviations: 5-ASA = 5-aminosalicylates, CD = Crohn disease, ECCO = European Crohn's and Colitis Organization, ITB = intestinal tuberculosis.

## References

[1] Bernell O Lapidus A Hellers G. Risk factors for surgery and recurrence in 907 patients with primary ileocaecal Crohn's disease. Br J Surg 2000;87:1697 701. 11122187 10.1046/j.1365-2168.2000.01589.x

[2] Bernstein CN Nabalamba A. Hospitalization, surgery, and readmission rates of IBD in Canada: a population-based study. Am J Gastroenterol 2006;101:110 8. 16405542 10.1111/j.1572-0241.2006.00330.x

[3] Burisch J Kiudelis G Kupcinskas L. Natural disease course of Crohn's disease during the first 5 years after diagnosis in a European population-based inception cohort: an Epi-IBD study. Gut 2019;68:423 33. 29363534 10.1136/gutjnl-2017-315568

[4] Chatu S Saxena S Subramanian V. The impact of timing and duration of thiopurine treatment on first intestinal resection in Crohn's disease: national UK population-based study 1989–2010. Am J Gastroenterol 2014;109:409 16. 24469612 10.1038/ajg.2013.462

[5] Chatu S Subramanian V Saxena S. The role of thiopurines in reducing the need for surgical resection in Crohn's disease: a systematic review and meta-analysis. Am J Gastroenterol 2014;109:23 34. quiz 5. 24322839 10.1038/ajg.2013.402

[6] Chou JW Lai HC Chang CH. Epidemiology and clinical outcomes of inflammatory bowel disease: a hospital-based study in central Taiwan. Gastroenterol Res Pract 2019;2019:4175923. 31312216 10.1155/2019/4175923 PMC6595318

[7] Chow DK Leong RW Lai LH. Changes in Crohn's disease phenotype over time in the Chinese population: validation of the Montreal classification system. Inflamm Bowel Dis 2008;14:536 41. 18058793 10.1002/ibd.20335

[8] Colombel JF Narula N Peyrin-Biroulet L. Management strategies to improve outcomes of patients with inflammatory bowel diseases. Gastroenterology 2017;152:351 61. e5. 27720840 10.1053/j.gastro.2016.09.046

[9] Das K Ghoshal UC Dhali GK. Crohn's disease in India: a multicenter study from a country where tuberculosis is endemic. Dig Dis Sci 2009;54:1099 107. 18770037 10.1007/s10620-008-0469-6

[10] Dignass A Van Assche G Lindsay JO. The second European evidence-based Consensus on the diagnosis and management of Crohn's disease: current management. J Crohns Colitis 2010;4:28 62. 21122489 10.1016/j.crohns.2009.12.002

[11] Gao X Yang RP Chen MH. Risk factors for surgery and postoperative recurrence: analysis of a south China cohort with Crohn's disease. Scand J Gastroenterol 2012;47:1181 91. 22845663 10.3109/00365521.2012.668931

[12] Hilmi I Jaya F Chua A. A first study on the incidence and prevalence of IBD in Malaysia: results from the Kinta Valley IBD Epidemiology Study. J Crohns Colitis 2015;9:404 9. 25744112 10.1093/ecco-jcc/jjv039

[13] Hwang SW Kim JH Im JP. Influence of age at diagnosis on the clinical characteristics of Crohn's disease in Korea: results from the CONNECT study. J Gastroenterol Hepatol 2017;32:1716 22. 28251684 10.1111/jgh.13775

[14] Jeuring SF van den Heuvel TR Liu LY. Improvements in the long-term outcome of Crohn's disease over the past two decades and the relation to changes in medical management: results from the population-based IBDSL cohort. Am J Gastroenterol 2017;112:325 36. 27922024 10.1038/ajg.2016.524

[15] Jiang L Xia B Li J. Retrospective survey of 452 patients with inflammatory bowel disease in Wuhan city, central China. Inflamm Bowel Dis 2006;12:212 7. 16534423 10.1097/01.MIB.0000201098.26450.ae

[16] Kalaria R Desai D Abraham P. Temporal change in phenotypic behaviour in patients with Crohn's Disease: do Indian patients behave differently from Western and other Asian patients? J Crohns Colitis 2016;10:255 61. 26519461 10.1093/ecco-jcc/jjv202 PMC4957468

[17] Kariyawasam VC Selinger CP Katelaris PH. Early use of thiopurines or methotrexate reduces major abdominal and perianal surgery in Crohn's disease. Inflamm Bowel Dis 2014;20:1382 90. 24991785 10.1097/MIB.0000000000000119

[18] Kim B Cheon JH Moon HJ. Crohn's disease prognosis and early immunomodulator therapy: results from the CONNECT study. J Gastroenterol Hepatol 2016;31:126 32. 26406334 10.1111/jgh.13169

[19] Kwak MS Kim DH Park SJ. Efficacy of early immunomodulator therapy on the outcomes of Crohn's disease. BMC Gastroenterol 2014;14:85. 24886458 10.1186/1471-230X-14-85 PMC4017088

[20] Lakatos PL Golovics PA David G. Has there been a change in the natural history of Crohn's disease? Surgical rates and medical management in a population-based inception cohort from Western Hungary between 1977–2009. Am J Gastroenterol 2012;107:579 88. 22233693 10.1038/ajg.2011.448

[21] Lee YJ Yang SK Byeon JS. Analysis of colonoscopic findings in the differential diagnosis between intestinal tuberculosis and Crohn's disease. Endoscopy 2006;38:592 7. 16673312 10.1055/s-2006-924996

[22] Li Y Chen B Gao X. Current diagnosis and management of Crohn's disease in China: results from a multicenter prospective disease registry. BMC Gastroenterol 2019;19:145. 31420025 10.1186/s12876-019-1057-2 PMC6697932

[23] Magro F Dias CC Coelho R. Impact of early surgery and immunosuppression on Crohn's disease disabling outcomes. Inflamm Bowel Dis 2017;23:289 97. 28107278 10.1097/MIB.0000000000001007

[24] Makharia GK Srivastava S Das P. Clinical, endoscopic, and histological differentiations between Crohn's disease and intestinal tuberculosis. Am J Gastroenterol 2010;105:642 51. 20087333 10.1038/ajg.2009.585

[25] Mokhtar NM Nawawi KNM Verasingam J. A four-decade analysis of the incidence trends, sociodemographic and clinical characteristics of inflammatory bowel disease patients at single tertiary centre, Kuala Lumpur, Malaysia. BMC Public Health 2019;19: (Suppl 4): 550. 31196184 10.1186/s12889-019-6858-2 PMC6565539

[26] Molodecky NA Soon IS Rabi DM. Increasing incidence and prevalence of the inflammatory bowel diseases with time, based on systematic review. Gastroenterology 2012;142:46 54. e42; quiz e30. 22001864 10.1053/j.gastro.2011.10.001

[27] Moon CM Park DI Kim ER. Clinical features and predictors of clinical outcomes in Korean patients with Crohn's disease: a Korean association for the study of intestinal diseases multicenter study. J Gastroenterol Hepatol 2014;29:74 82. 23981141 10.1111/jgh.12369

[28] Ng SC Leung WK Shi HY. Epidemiology of inflammatory bowel disease from 1981 to 2014: results from a territory-wide population-based registry in Hong Kong. Inflamm Bowel Dis 2016;22:1954 60. 27416041 10.1097/MIB.0000000000000846

[29] Ng SC Tang W Ching JY. Incidence and phenotype of inflammatory bowel disease based on results from the Asia-pacific Crohn's and colitis epidemiology study. Gastroenterology 2013;145:158 65. e2. 23583432 10.1053/j.gastro.2013.04.007

[30] Ng SC Zeng Z Niewiadomski O. Early course of inflammatory bowel disease in a population-based inception cohort study from 8 countries in Asia and Australia. Gastroenterology 2016;150:86 95. e3; quiz e13-4. 26385074 10.1053/j.gastro.2015.09.005

[31] Nguyen GC Nugent Z Shaw S. Outcomes of patients with Crohn's disease improved from 1988 to 2008 and were associated with increased specialist care. Gastroenterology 2011;141:90 7. 21458455 10.1053/j.gastro.2011.03.050

[32] Oh EH Oh K Han M. Early anti-TNF/immunomodulator therapy is associated with better long-term clinical outcomes in Asian patients with Crohn's disease with poor prognostic factors. PLoS One 2017;12:e0177479. 28542298 10.1371/journal.pone.0177479 PMC5441601

[33] Pang P Ng YS Sidhu J. Epidemiology of inflammatory bowel disease in southern Peninsular Malaysia. Med J Malaysia 2018;73:86 9. 29703871

[34] Park SH Yang SK Park SK. Long-term prognosis of crohn's disease and its temporal change between 1981 and 2012: a hospital-based cohort study from Korea. Inflamm Bowel Dis 2014;20:488 94. 24412992 10.1097/01.MIB.0000441203.56196.46

[35] Peyrin-Biroulet L Harmsen WS Tremaine WJ. Surgery in a population-based cohort of Crohn's disease from Olmsted County, Minnesota (1970–2004). Am J Gastroenterol 2012;107:1693 701. 22945286 10.1038/ajg.2012.298 PMC3572861

[36] Present DH Korelitz BI Wisch N. Treatment of Crohn's disease with 6-mercaptopurine. A long-term, randomized, double-blind study. N Engl J Med 1980;302:981 7. 6102739 10.1056/NEJM198005013021801

[37] Punati J Markowitz J Lerer T. Effect of early immunomodulator use in moderate to severe pediatric Crohn disease. Inflamm Bowel Dis 2008;14:949 54. 18306311 10.1002/ibd.20412

[38] Qiu Y Chen BL Feng R. Prolonged azathioprine treatment reduces the need for surgery in early Crohn's disease. J Gastroenterol Hepatol 2018;33:664 70. 28940780 10.1111/jgh.14000

[39] Qiu Y Chen BL Mao R. Early thiopurines versus conventional step-care therapy for modifying the disease course of early Crohn's disease: a tertiary referral center cohort study. Medicine (Baltimore) 2015;94:e1148. 26252273 10.1097/MD.0000000000001148 PMC4616617

[40] Ramadas AV Gunesh S Thomas GA. Natural history of Crohn's disease in a population-based cohort from Cardiff (1986–2003): a study of changes in medical treatment and surgical resection rates. Gut 2010;59:1200 6. 20650924 10.1136/gut.2009.202101

[41] Ronnblom A Holmstrom T Karlbom U. Clinical course of Crohn's disease during the first 5 years. Results from a population-based cohort in Sweden (ICURE) diagnosed 2005–2009(). Scand J Gastroenterol 2017;52:81 6. 27632773 10.1080/00365521.2016.1230777

[42] Rungoe C Langholz E Andersson M. Changes in medical treatment and surgery rates in inflammatory bowel disease: a nationwide cohort study 1979–2011. Gut 2014;63:1607 16. 24056767 10.1136/gutjnl-2013-305607

[43] Safroneeva E Vavricka SR Fournier N. Impact of the early use of immunomodulators or TNF antagonists on bowel damage and surgery in Crohn's disease. Aliment Pharmacol Ther 2015;42:977 89. 26271358 10.1111/apt.13363

[44] Sato Y Matsui T Yano Y. Long-term course of Crohn's disease in Japan: incidence of complications, cumulative rate of initial surgery, and risk factors at diagnosis for initial surgery. J Gastroenterol Hepatol 2015;30:1713 9. 26094852 10.1111/jgh.13013

[45] Silverberg MS Satsangi J Ahmad T. Toward an integrated clinical, molecular and serological classification of inflammatory bowel disease: report of a Working Party of the 2005 Montreal World Congress of Gastroenterology. Can J Gastroenterol 2005;19: (suppl A): 5A 36A. 10.1155/2005/269076 16151544

[46] To N Gracie DJ Ford AC. Systematic review with meta-analysis: the adverse effects of tobacco smoking on the natural history of Crohn's disease. Aliment Pharmacol Ther 2016;43:549 61. 26749371 10.1111/apt.13511

[47] Wei SC Lin MH Tung CC. A nationwide population-based study of the inflammatory bowel diseases between 1998 and 2008 in Taiwan. BMC Gastroenterol 2013;13:166. 24314308 10.1186/1471-230X-13-166 PMC4028859

[48] Ye BD Yang SK Cho YK. Clinical features and long-term prognosis of Crohn's disease in Korea. Scand J Gastroenterol 2010;45:1178 85. 20560811 10.3109/00365521.2010.497936

[49] Yen HH Weng MT Tung CC. Epidemiological trend in inflammatory bowel disease in Taiwan from 2001 to 2015: a nationwide populationbased study. Intest Res 2019;17:54 62. 30449079 10.5217/ir.2018.00096 PMC6361021

[50] Zhulina Y Udumyan R Tysk C. The changing face of Crohn's disease: a population-based study of the natural history of Crohn's disease in Orebro, Sweden 1963–2005. Scand J Gastroenterol 2016;51:304 13. 26448101 10.3109/00365521.2015.1093167

